# A Decrease in Sex Ratio at Birth Nine Months after the Earthquake in L'Aquila

**DOI:** 10.1100/2012/162017

**Published:** 2012-06-18

**Authors:** A. D'Alfonso, F. Patacchiola, I. Colagrande, G. D'Alessandro, A. Di Fonso, P. Palermo, G. Carta

**Affiliations:** ^1^Department of Gynecology and Obstetrics, San Salvatore Hospital, L'Aquila, Italy; ^2^Department of Health Sciences, University of L'Aquila, 67100 L'Aquila, Italy

## Abstract

*Introduction*. Multiple factors influence the secondary sex ratio (SSR) including stress, which appears to affect mainly the males born. *Objective*. We evaluate the effects of the earthquake in L'Aquila on the SSR. *Materials and Methods*. The SSR for the first six months of 2010 was compared to that of the same period of 2008. The chi-square test and Fisher's test were used for the statistical analysis. *Results*. Nine months after the earthquake, an important reduction in the SSR was recorded: January 2010 versus January 2008 =0.62 versus 0.96. An overall fall in the SSR was also recorded when the first 3 months of 2010 were compared to the first three months of 2008: 0,82 versus 1,11. When the first three months of 2010 were compared with the second three months of 2010, a statistically significant increase of the sex ratio at birth was noted (0,82 versus 1,27).

## 1. Introduction

In the human species the ratio between males and females at birth, the secondary sex ratio (SSR), is not 50 : 50 as would be expected given the equal number of X and Y spermatozoa, but slightly biased towards the male sex. Worldwide, the average value of the SSR is 1.07 in favour of the male sex indicating that other factors influence this ratio [1].

## 2. Selection of the Sex of the Unborn Child

Scientific literature has shown that the sex of an unborn child can be influenced by a number of factors (hormonal factors, parents' age, pollution, nutritional and energetic factors, stress) that, in the preconception phase, can affect the penetration of either the X or the Y spermatozoon and, in the postconception phase, determine the negative selection of the conceived male or female child.

In particular, both physical and psychological stress influences fertility exercising a negative effect in the very early stages of pregnancy resulting in miscarriage, notably of male fetuses [2]. Stress may also influence the ratio of males to females at conception, the Primary Sex Ratio (PSR), as a result of its effect on preconception factors such as hormonal state, sexual activity, and seminal liquid quality. Natural catastrophes also represent a source of intense acute stress: M. Fukuda et al. noted a fall in the number of male infants born nine months after the earthquake that hit the city of Kobe in 1995 [3]; this fall was associated with a reduction in the motility of spermatozoa [4]. Similarly, a decline in the sex ratio was reported in a study carried out after the earthquake in the city of Bam, in Iran in 2003 [5]. Further stressful events include terrorism and war: a decline in the SSR, as a result of a fall in the conception of males, and an increase in the number of male fetal deaths following the terrorist attack on the twin towers on 11th September 2001 was observed [6]. The war in Slovenia in 1991, which only lasted 10 days, resulted in a temporary fall in the level of sex hormones and sexual activity, as well as the quality and mobility of spermatozoa; this was reflected in the fall in the sex ratio recorded for Lubiana, the capital and target of consistent bombing [7]. The war in Iran and Iraq (1980–1988) also resulted in a fall in the number of males born but in this case other factors may have influenced the sex ratio such as chemical warfare [8]. 

All these findings support a greater susceptibility of males to stressful events during pregnancy.

## 3. The Greater Vulnerability of Males during Pregnancy and at Birth

The primary sex ratio is not 100/100 but biased towards males. However, during pregnancy, both in humans and other species, there is a greater loss of male embryos and fetuses. At the end of the second month of pregnancy the ratio of male to females is 151/100; this falls to 132/100 by the end of the third month [9] and continues to fall until birth when the average ratio is 107/100 [1, 10]. It would therefore appear that a higher number of male embryos are conceived to compensate for the greater mortality of the male sex as a result of their greater susceptibility to adverse and stressful events. In fact spontaneous miscarriages, even in the absence of chromosomal abnormalities, tend to be of male embryos or fetuses [11, 12]; moreover an intensive neonatal care is more frequently required for male infants according to lower Apgar scores 1 to 5 minutes [13], and perinatal mortality is greater in males than in females both in end term pregnancies and in preterm pregnancies [13–15]. Similarly it would appear that the SSR at birth is still biased towards the male sex to compensate for higher mortality during infancy, childhood, and adult life which only equals out towards the fourth decade of life. The mortality at one year of life is 5,4% in males versus 4,1% in females [12].

## 4. The Earthquake in L'Aquila

On 6th of April 2009, at 3:32 am, an earthquake of magnitude 5.9 on the Richter Scale hit the region of L'Aquila; 308 people died and 1600 were injured of whom 200 seriously. Patients were taken to hospitals in the surrounding areas as the city's main hospital had to be evacuated. The earthquake destroyed approximately 48% of residential property, 21.2% of public property, and 53.7% of the city's cultural patrimony. The entire population was evacuated, and a significant proportion of people lost their jobs. The government declared a state of emergency, and 67,459 people required assistance and accommodation in the following months. Two hundred and fifty aftershocks a day were recorded for the entire month of April; the aftershocks fell to 40–60 in May and continued in the following months [16]. An earthquake, like other catastrophes, is a source of acute stress, and the aim of this study was to evaluate its impact on the SSR.

## 5. Materials and Methods

On the basis of the fact that the children conceived after 6th April 2009 would be born in January 2010, we studied and compared births from the 1st of January to June 30, 2010 (800 born) and for comparison purposes from the 1st of January to June, 30, 2008 (1026 born), using the records from the Clinical Obstetrics and Gynaecology Department of the San Salvatore Hospital, L'Aquila. The chi-square test and Fisher's test were used for the statistical analysis.

## 6. Results

**Figure 1:**
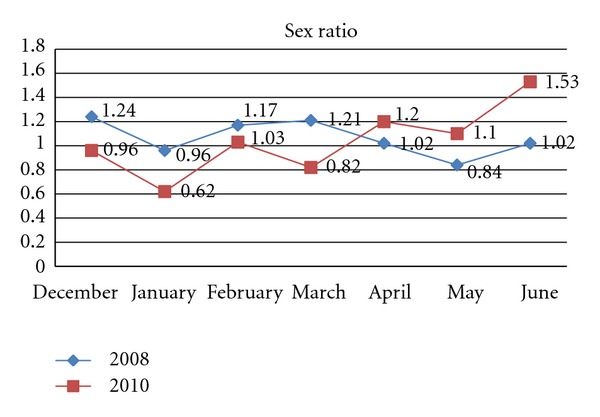
Sex ratio trends using the chi-squared test *P* = 0.22.

Nine months after the earthquake an important reduction in the secondary sex ratio at birth was recorded, although the overall fall was not statistically significant: the SSR for January 2010 was 0,62, whilst a value of 0,96 was recorded for the same month in 2008 (*P* = 0.23). The percentage of males and females born in January 2010 was, respectively, 38,30% and 61,70%, whilst for January 2008 these figures were 48,91% and 51,09%, respectively. A fall in the sex ratio (0,82 versus 1,11) (*P* = 0.13) was also observed when births over three months were compared: the first trimester of 2010 (January, February and March) compared to the same period of 2008. Statistically significant differences in the sex ratio were recorded when the first trimester of 2010 was compared to the second: 0,82 versus 1,27 (*P* = 0.031). Lastly, variations in the SSR over the two years taken into consideration were compared using the chi-squared test for trend; however no statistically significant findings emerged (*P* = 0.22) (Figure 1). 

## 7. Discussion

This study highlights a fall in the percentage of males born nine months after the earthquake in L'Aquila: in January 2010 the SSR was approximately 0.62, much lower than the global average value of 1.07. These findings echo those of M. Fukuda et al. concerning the earthquake in Kobe [3] and confirm clinical evidence pointing to a greater vulnerability of the male sex at conception and during embryo-fetal development, as well as following acute stress. We also found that the sex ratio returned to its expected values six months after the earthquake. In fact after the initial fall in male births, there was an inversion of the trend and the value of the SSR which, from its lowest value of 0.62 in January, rose steadily to reach a peak of 1.53 in June. This statistically significant variation underlines how catastrophic events can determine changes in the SSR in favour of the female sex especially in the immediate term. In fact the adverse effects of acute stress are mostly felt in the first months of pregnancy which are also the most vulnerable period in biological terms and the trimester in which the highest number of miscarriages occur. It has been suggested that, vice versa, as pregnancy proceeds, the woman and her child are less susceptible to the effects of stress [17].
